# Changes in glial cell phenotypes precede overt neurofibrillary tangle formation, correlate with markers of cortical cell damage, and predict cognitive status of individuals at Braak III-IV stages

**DOI:** 10.1186/s40478-022-01370-3

**Published:** 2022-05-09

**Authors:** Raquel N. Taddei, Maria V. Sanchez-Mico, Orla Bonnar, Theresa Connors, Angelica Gaona, Dominique Denbow, Matthew P. Frosch, Teresa Gómez-Isla

**Affiliations:** 1 Department of Neurology, Massachusetts General Hospital, 15th Parkman St, Boston, MA 02114 USA; 2 Massachusetts Alzheimer’s Disease Research Center, Boston, MA USA; 3 Department of Neurology, Dementia Research Institute, University College London, London, UK; 4 C.S. Kubik Laboratory for Neuropathology, Massachusetts General Hospital, Boston, MA USA

**Keywords:** AD, Neurofibrillary tangles, Glial phenotypes, Cortical cell vulnerability, White matter changes, Cognition

## Abstract

Clinico-pathological correlation studies show that some otherwise healthy elderly individuals who never developed cognitive impairment harbor a burden of Alzheimer’s disease lesions (plaques and tangles) that would be expected to result in dementia. In the absence of comorbidities explaining such discrepancies, there is a need to identify other brain changes that meaningfully contribute to the cognitive status of an individual in the face of such burdens of plaques and tangles. Glial inflammatory responses, a universal phenomenon in symptomatic AD, show robust association with degree of cognitive impairment, but their significance in early tau pathology stages and contribution to the trajectory of cognitive decline at an individual level remain widely unexplored. We studied 55 brains from individuals at intermediate stages of tau tangle pathology (Braak III-IV) with diverging antemortem cognition (demented vs. non-demented, here termed `resilient’), and age-matched cognitively normal controls (Braak 0-II). We conducted quantitative assessments of amyloid and tau lesions, cellular vulnerability markers, and glial phenotypes in temporal pole (Braak III-IV region) and visual cortex (Braak V-VI region) using artificial-intelligence based semiautomated quantifications. We found distinct glial responses with increased proinflammatory and decreased homeostatic markers, both in regions with tau tangles (temporal pole) and without overt tau deposits (visual cortex) in demented but not in resilient. These changes were significantly associated with markers of cortical cell damage. Similar phenotypic glial changes were detected in the white matter of demented but not resilient and were associated with higher burden of overlying cortical cellular damage in regions with and without tangles. Our data suggest that changes in glial phenotypes in cortical and subcortical regions represent an early phenomenon that precedes overt tau deposition and likely contributes to cell damage and loss of brain function predicting the cognitive status of individuals at intermediate stages of tau aggregate burden (Braak III-IV).

## Introduction

The frequency of both amyloid plaques and neurofibrillary tangles (the classic neuropathologic hallmarks of AD) and dementia increases with age [14, 26, 72]. Yet, multiple clinicopathological correlation studies have shown that amyloid plaques and neurofibrillary tangles (NFTs) do not suffice to fully explain the extent of brain injury responses (e.g. neuronal and synaptic loss) observed in AD brains [3, 20, 28, 87] nor do inevitably lead to cognitive impairment in all individuals [18, 21, 73, 77]. In cohort studies of aging and dementia that include brain donation, about 5–7% of brains from older people without cognitive impairment demonstrate a high stage of tau tangle pathology (Braak V-VI), and up to 45% exhibit an intermediate stage (Braak III-IV) at postmortem (reviewed in [79]). In agreement with these observations, up to 30% of cognitively unimpaired elderly volunteers are amyloid-positive in PET imaging studies [1, 42, 54, 59, 75], and around 13% are tau-positive by PET as well [9]. Moreover, recent studies have highlighted that the vast majority of post-mortem brains of individuals 85 years of age and older show some degree of classic AD neuropathologic changes, which by large exceeds the expected antemortem prevalence of dementia in these and subsequent age groups which is cumulatively estimated to be at around 50% [10, 52, 53].The phenomenon of absence of overt cognitive deterioration in the face of neuropathologic lesions expected to have had a deleterious impact on cognition is the process we and others have termed 'resilience' to AD pathology [5, 68]. The discrepancy remains even after adjusting for the presence of other age-related common co-morbidities such as TDP-43 and alpha-synuclein aggregates or vascular lesions that also contribute to clinical disease expression [5, 68]. These observations from resilient brains suggest that additional mechanisms beyond plaques and tangles must be the real culprits driving the tissue injury responses (e.g. neuronal and synaptic loss) that ultimately result in brain functional changes and impaired cognition in AD, and may contribute to the wide inter-individual heterogeneity in the clinical expression of amyloid and tau containing lesions.

Detailed examination of high tangle burden (Braak V-VI) demented and resilient brains by our group and others has shown that one of the most robust and significant differences is the presence of substantially increased glial responses, comprising inflammatory microglia and astrocytes [56, 68], alongside a distinct proinflammatory cytokine expression profile in the demented [5]. Glial involvement in AD pathogenesis has only recently gained considerable interest fueled by genome-wide association studies (GWAS) that identified risk loci in genes related to the immune system in human [7, 30, 31, 34, 51, 98] along with novel insights of the unexpected involvement of microglia in synaptic pruning and modulation of synaptic activity in mouse models [35, 86, 96] and in the human AD brain [89]. Importantly, brain glial responses are present early and increase around amyloid plaques and tau tangles with progression of symptoms in clinically manifest AD [80].

Emerging studies are highlighting the broad heterogeneity in phenotypes of astrocytes and microglial cells in AD but the role of each of these glial phenotypes (e.g. pro-inflammatory and homeostatic) in the clinical expression of plaques and tangles and other common neurodegenerative lesions, and in the tissue injury responses (synaptic and neuronal damage) and trajectory of cognitive impairment are incompletely understood [4, 44, 78, 82, 83]. Some studies have suggested that increased proinflammatory glia in early mild cognitive impairment (MCI) stages may exert a beneficial role on preserving brain structure and function [20], while identical phenotypes in subsequent phases of the dementing disorder are associated with increased severity and faster rates of cognitive dysfunction [37, 57, 65]. This suggests that glial phenotypes may follow a non-linear trajectory and likely switch their function during disease progression [24]. Recent data in animal models of AD and human AD brains have pointed to the potentially even more relevant loss of homeostatic glial markers early in the disease process and to their correlation with the extent of synaptic loss [33, 62, 85, 91].

In addition, recent evidence from neuroimaging and autopsy studies has suggested that white matter (WM) damage comprising axonal loss, astrogliosis, and oligodendrocyte dysfunction accounts for one of the earliest pathologic changes that may be implicated in the risk and progression of AD [23, 62]. Early axonal dysfunction and WM lesions [49] have both been associated with a higher burden of overlying cortical AD lesions [58], and microstructural WM changes have been correlated with the degree of cognitive impairment in AD [17], with imaging studies indicating that significant loss of WM volume and axonal connectivity is present very early from MCI stages of the condition [48]. Moreover, in familial early-onset AD mutation carriers, functional and structural changes in the WM have been found 5–10 years prior to dementia onset and have been associated with increased pro-inflammatory microglial markers in these individuals [2]. Postmortem studies in sporadic late-onset AD have endorsed that inflammatory changes in WM occur early [70], and in vivo PET imaging studies suggest that microglial inflammation in WM is associated with higher burden of small vessel disease markers (WM hyperintensities) and cognitive impairment [55].

Whether an aberrant activation and/or lack of homeostatic properties of glial elements in cortical and juxtacortical WM regions substantially contribute to neuronal damage and loss of brain function in AD or reflect a mere response to neuronal injury remains controversial. The study of brains at intermediate stages of tau burden (Braak III-IV) with divergent ante-mortem cognitive status (demented vs. resilient) provides a valuable opportunity to assess early presence and interaction(s) between glial cell responses and brain changes other than Aβ and tau that occur in regions yet devoid of NFTs and that could be relevant to the earliest and most preventable phases of neurodegeneration in AD. We hypothesized that changes in cortical and subcortical glial phenotypes precede overt NFT formation and correlate with early markers of cortical cell damage predicting the cognitive fate of individuals at intermediate stages of tau burden (Braak III-IV).

## Material and methods

### Human brain samples

The study included 55 cases from the Massachusetts General Hospital ADRC Brain Bank. Cases were scored by Thal phases for Aβ deposition (0–5) [88], Braak stage for NFTs (0–VI) [13], and the Consortium to Establish a Registry for Alzheimer’s Disease (CERAD) for neuritic plaques (A-C) [60], and divided into three groups: (1) non-demented individuals before death whose post-mortem exam demonstrated a Braak stage 0-II (controls) (n = 8); (2) non-demented individuals before death whose post-mortem examination demonstrated a Braak stage III-IV (resilient) (n = 20); and (3) demented individuals before death whose post-mortem examination demonstrated a Braak stage III-IV (demented) (n = 27) [13].

The majority of both demented and resilient brains in these series also harbored intermediate loads of amyloid neuritic plaques (moderate CERAD scores) meeting criteria for an intermediate probability of AD at autopsy according to NIA-AA guidelines [39]. A smaller subset of brains with tangle Braak stage III-IV and a CERAD neuritic plaque score `none’ but different cognitive outcomes (impaired vs. cognitively normal `resilient’) meeting criteria for primary age-related tauopathy (PART) [19] were also included in the demented and resilient group categories (n = 3 and 5, respectively). NFTs in PART are indistinguishable from those of AD, in the absence of amyloid beta (Aβ) plaques, and symptoms in these individuals usually range from normal to amnestic cognitive changes. In order to align with the Braak stage III-IV tangle burden of the cases with plaques, these cases of presumed PART had only mesial temporal lobe involvement. Of all cases, 24 (including 4 controls, 6 resilient and 14 demented) had undergone a formal cognitive evaluation within 2 years of death including measures of attention, processing speed, executive function, episodic memory and language, as part of their longitudinal enrollment in the Uniform Data Set (UDS) of the Alzheimer’s Disease Centers (ADC) program of the National Institute on Aging (NIA) [97]. The rest were individuals with either no reported dementia or dementia according to their clinical records and death certificates. Histological evaluation was performed using a set of regional tissue blocks representative of the spectrum of neurodegenerative diseases. All blocks were routinely stained with Luxol fast blue, haematoxylin, and eosin and selected blocks were stained for Bielschowsky silver stain and Aβ, phospho-TDP-43, alpha-synuclein, ubiquitin and phospho-tau immunoreactivity. Neuropathological diagnosis was recorded, and cases with evidence of cortical Lewy body pathology, phospho-TDP-43 aggregates, or other lesions different to classic AD pathology were excluded. The human demographics, neuropathological data and Mini-Mental State Exam (MMSE) scores of the cases prior to death are summarized in Table 1. The three groups were carefully matched for age, gender, co-morbidities, as well as additional systemic factors like terminal septicemia or prolonged hypoxia. Resilient and demented groups were matched for Thal phase, Braak stage, CERAD neuritic plaque score, ApoE genotype and postmortem intervals (PMIs). A composite vascular score was also calculated based on hypertensive cerebrovascular, atherosclerosis, occlusive atherosclerosis and cerebral amyloid scores as evaluated by neuropathologists on a rating scale from 0–3 (none, mild, moderate, severe) [94]. All autopsies were performed according to standardized protocols [93] and tissue collection and use was approved by the local Institutional Review Boards.

**Table 1:** Baseline demographic and neuropathological features of the total N = 55 subjects included

Cognitive status	Control	Resilient	Demented
Number of subjects, total N = 55	8	20	27
Mini-mental state exam			
Mean (SD)	29 (0.8)	29.1 (0.9)	23.7 (6.6)
Age (years)			
Mean (SD)	82.6 (8.3)	88.9 (9)	88.1 (6.1)
Gender			
Female N (%)	4 (50%)	11 (55%)	13 (48%)
Male N (%)	4 (50%)	9 (45%)	14 (52%)
ApoE allele status			
ApoE2 allele (%)	0 (0%)	2 (9%)	2 (7%)
ApoE3 allele (%)	8 (100%)	18 (82%)	22 (79%)
ApoE4 allele (%)	0 (0%)	2 (9%)	4 (14%)
Neuropathology ('ABC' score)			
A-Thal phase, range (mean)	A0-4 (1)	A0-4 (2.2)	A0-5 (2.2)
B-Braak stage, range (mean)	0-II (1.4)	III-IV (3.5)	III-IV (3.5)
C-CERAD score, range (mean)	C0-2 (0.4)	C0-3 (1.4)	C0-3 (1.2)
Vascular score			
Composite score, mean (SD)*	3.8 (2)	3 (1.9)	5 (2.4)
PMI (hours)			
Mean (SD)	24.5 (22.8)**	19.8 (11.8)	17.7 (9.4)**

* Thal phase: No amyloid deposition (A0), amyloid in neocortex (A1), amyloid in allocortex/limbic region (A2), amyloid in diencephalon/basal ganglia (A3), amyloid in brainstem/midbrain (A4), amyloid in cerebellum (A5); ** CERAD score: No neuritic plaques (C0), sparse plaques (C1), moderate plaques (C2), frequent plaques (C3); *** Cerebrovascular composite score includes subscores for: hypertensive cerebrovascular, atherosclerosis, cerebral atherosclerosis, occlusive atherosclerosis and cerebral amyloid score. *PMI* Postmortem interval; *SD* Standard deviation

### Semi-automated quantitative neuropathology using artificial intelligence-based analyses

Quantitative neuropathological assessments were derived from two regions of interest, temporal pole (Brodmann’s area 38) and visual cortex (Brodmann’s area 17/18), representing regions known to be affected by NFT pathology at Braak stages III-IV and V-VI respectively. Brain tissue sections derived from formalin-fixed paraffin-embedded tissue blocks were cut at a thickness of 7 µm. Immunohistochemical staining was conducted using a Bond RX autostainer from Leica Biosystems. We used primary antibodies to label Aβ deposits (4G8, Bio Legend, 1:8000), phospho-tau (AT8, Thermo Fisher, 1:500), phospho-TDP43 (Fisher Scientific, 1:3000), alpha-synuclein (Fisher Scientific, 1:200), cellular DNA damage (γH2AX, Abcam, 1:1000), constitutive and inflammatory astrocytic markers (ALDH1L1, Abcam, 1:200; GFAP, Sigma Aldrich, 1:20,000), constitutive oligodendrocyte markers (Olig2, Abcam, 1:100), and constitutive, inflammatory and homeostatic microglial markers (IBA1, Abcam, 1:500; CD68, Dako, 1:500; HLA-DR, Abcam, 1:50; TMEM119, Sigma Aldrich, 1:50; P2RY12, Fisher Scientific, 1:100) followed by secondary anti-mouse and anti-rabbit horseradish peroxidase (HRP) antibody conjugation (BOND Polymer Refine Detection, catalog number DS9800, Leica Biosystems Newcastle, UK) following the Leica autostainer protocol. Immunolabeling was visualized using 3,3'-diaminobenzidine (DAB) and slides were counterstained with the nuclear marker haematoxylin using the Leica Biosystems autostainer protocol. Slides were manually dehydrated, cover-slipped using Permount mounting medium and scanned with a Nanozoomer XR (NDP.scan 3.3.3, Hamamatsu Photonics, Hertfordhsire, UK) using a 20X objective.

**Fig. 1:**
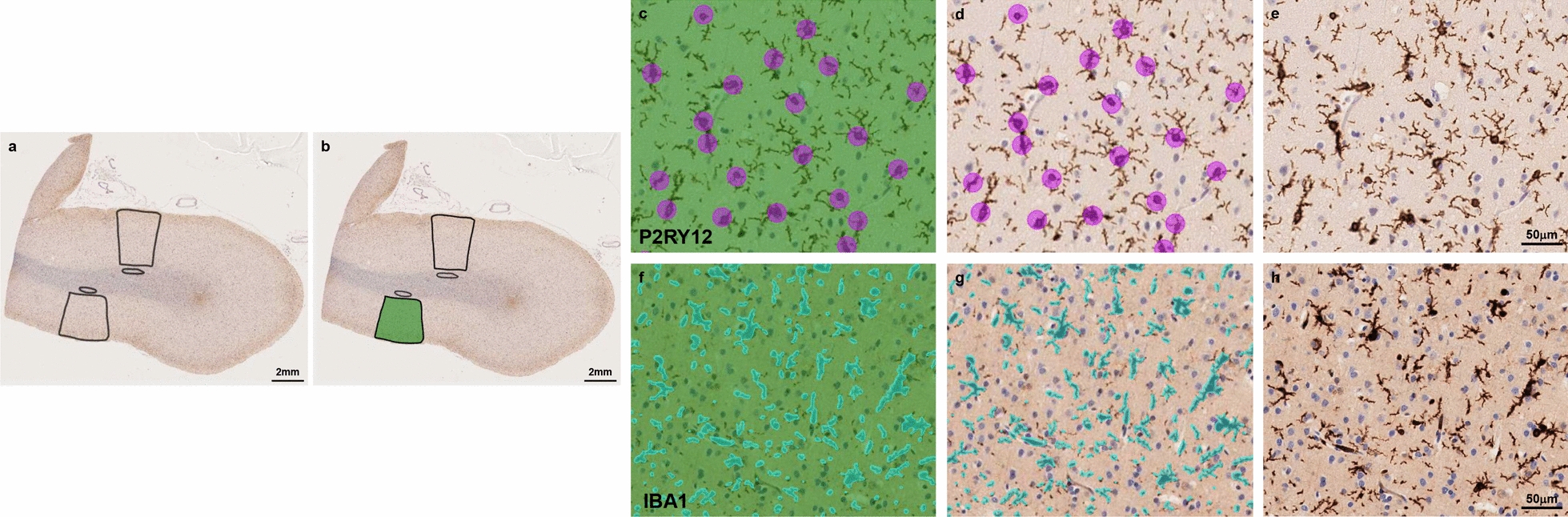
Example of the artificial intelligence based semiautomated quantification method used. Selection of cortical and subcortical regions of interest (**a**, **b**), tissue layer detection in green (**b**, **c**, **f**), P2RY12 counter in purple (**c**, **d**), IBA1 area detector in blue (**f**, **g**) and unannotated images stained with P2RY12 (**e**) and IBA1 (**h**)

To quantify positively labelled cells and lesions, we used an artificial intelligence (AI) based semi-automated quantification method (Aiforia version 5.2, Aiforia Inc, Cambridge, MA), which allowed us to train 2-dimensional AI models, perform human-based validation(s) and apply individual AI models for accurate cell and lesion quantification(s) in predetermined regions of interest. To this end, scanned slides were uploaded to the Aiforia cloud-based platform and stored in folders. Two regions of interest (ROIs) were selected for each brain slide in cortical and adjacent subcortical brain regions respectively. Cortical ROIs were determined based on representative and well-preserved brain regions containing the 6 cortical layers and measuring a total of 6 mm in length along the pial surface (Fig. 1a, b). Subcortical ROIs were selected in the juxtacortical WM immediately adjacent to the cortical ROI region and were drawn as circular or oval sections, avoiding inclusion of major vessels or gross artifacts (Fig. 1a, b). Two ROIs were selected for each individual in cortical and adjacent subcortical brain regions respectively. Total number of cells and lesions, average density (cells or lesions/mm^2^), and lesion burdens (percentage of cortex and WM occupied by lesions) were quantified within the sum of the two cortical and subcortical ROIs (representative examples of AI model performance for P2RY12 and IBA1 stainings are shown in Fig. 1c–h).

Aiforia-based AI models and quantifications were performed in the cortex for the antibodies 4G8, AT8, γH2AX, ALDH1L1, GFAP, IBA1, CD68, HLA-DR, TMEM119 and P2RY12 and in subcortical WM for Olig2, IBA1, CD68, HLA-DR, TMEM119 and P2RY12. Models were created to quantify `objects’ (positive cells) for AT8, γH2AX, ALDH1L1, GFAP, CD68, HLA-DR, TMEM119 and P2RY12 antibodies, and `burdens’ (% of cortex/juxtacortical WM labelled by the antibody of interest) for 4G8 and IBA1. No AI model for formal quantification of alpha synuclein or pTDP43 was performed since absence of those comorbidities was a prerequisite when selecting the brains included in the present study.

To better assess colocalization of γH2AX with glial markers (IBA1 and GFAP) with increased resolution at the cellular level, we expanded human brain sections following a previously published expansion microscopy (ExM) protocol [16]. We used primary antibodies to label IBA1 (Synaptic Systems, 1:100), GFAP (G3893, 1:100) and γH2AX (Abcam, 1:100), followed by the appropriate secondary antibodies (Alexa Fluor 488, 555, and 647, 1:100). It has been previously demonstrated that the ExM technique attains increased epitope exposure and isotropic tissue magnification, substantially increasing image resolution to about 4-5X [27].

A summary of the antibodies used in this study is displayed in Table 2.

**Table 2:** List of antibodies used in the study

Antibody	Company	Serial number	1° host	Clonality	Dilution
AT8	Thermo Fisher Scientific	MN1020	Mouse	Monoclonal	1:500
4G8	Bio Legend	800709	Mouse	Monoclonal	1:8000
H2AX	Abcam	ab26350	Mouse	Monoclonal	1:1000
ALDH1L1	Abcam	ab177463	Rabbit	Monoclonal	1:200
GFAP	Sigma aldrich	G3893	Mouse	Monoclonal	1:20,000
Olig2	Abcam	ab109186	Rabbit	Monoclonal	1:100
IBA1	Abcam	ab178847	Rabbit	Monoclonal	1:500
CD68	Dako	M0814	Mouse	Monoclonal	1:500
HLA-DR	Abcam	ab7856	Mouse	Monoclonal	1:50
TMEM119	Sigma aldrich	HPA051870	Rabbit	Polyclonal	1:50
P2RY12	Thermo Fisher Scientific	NBP233870	Rabbit	Polyclonal	1:100
**Antibody**	**Company**	**Serial number**	**1° host**	**Clonality**	**Dilution**
GFAP	Sigma Aldrich	G3893	Mouse	Monoclonal	1:100
IBA1	Synaptic Systems	234009	Chicken	Monoclonal	1:100
γH2AX	Abcam	ab81299	Rabbit	Monoclonal	1:100

### Statistical analyses

D’Agostino-Pearson normality test was applied to test for Gaussian distribution. Multiple group analyses were then performed using one-way ANOVA for parametric variables and Kruskall-Wallis for non-parametric variables. Posthoc analyses to assess for between group differences were evaluated with Holm-Šídák’s test.

Correlation analyses were performed using Pearson test when both variables were normally distributed and Spearman test when at least one of the variables was not normally distributed. Significance level was set at *p* < 0.05. All statistical analyses and graphs were generated using Graphpad Prism version 9.3.0 (Graphpad Software Inc, La Jolla, CA).

Data are presented as mean ± standard error, unless otherwise indicated.

## Results

Regional Aβ-plaque burden and number of neurofibrillary tangles did not show significant differences between resilient and demented brains at Braak III-IV stages.

Recent studies have stressed that regional burden of amyloid plaques and NFTs may be a better predictor of cognition than the mere presence or absence of those lesions [52]. It is well known that amyloid burden increases in a linear fashion over a period of up to 15 years in advance of clinical symptoms before reaching a plateau [41]. Tangles, however, continue to accumulate linearly within brain regions as tau pathology progresses along well-defined tau spreading pathways (from Braak I-II, transentorhinal/entorhinal cortex, to Braak VI, primary visual cortex) correlating with the severity of cognitive decline and the amount of neuronal loss in symptomatic individuals [32, 40, 41, 90].

**Fig. 2:**
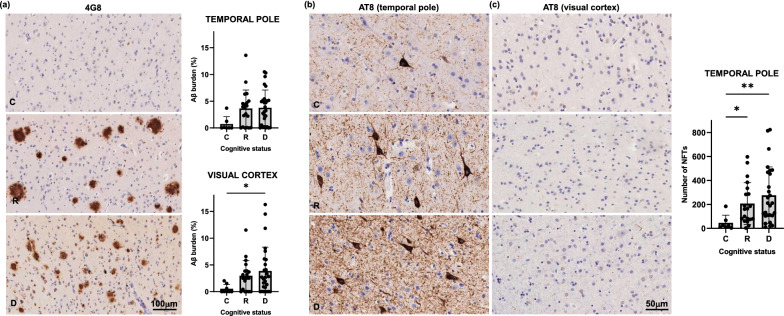
Regional Aβ plaque burden (defined as the percentage of cortex occupied by amyloid beta (Aβ) deposits immunostained with 4G8 antibody) (**a**) and number of neurofibrillary tangles (as reported by immunolabeling with AT8 antibody) (**b**, **c**) did not significantly differ in demented compared to resilient. Representative photomicrographs of plaques (**a**) and tangles (**b**, **c**) on sections from control, resilient and demented cases containing temporal pole (**a** and **b**) and visual cortex (**c**). *C* Control (Braak 0-II); *R* Resilient (Braak III/IV); *D* Demented (Braak III/IV); *p < 0.05; **p < 0.01. Scale bars 100 μm and 50 μm

Thus, even though demented and resilient brains were well matched for Thal phases, Braak stages, and CERAD scores, we sought to carefully quantify Aβ plaque and tau NFT load in the ROIs. Aβ-plaque burden (defined as the percentage of cortex in the ROIs occupied by deposits immunolabeled with 4G8 antibody) did not show statistically significant differences between resilient and demented brains in either temporal pole or visual cortex, suggesting that neither plaque scores nor regional Aβ-plaque loads were predictive of the different cognitive fate of those two groups (Fig. 2a). Quantification of number of NFTs (as visualized by immunolabeling with AT8 antibody) in the temporal pole did not show significant differences between resilient and demented either (*p* = 0.33), even though a trend towards higher NFT counts was observed in this region in the demented group (Fig. 2b). As expected at tangle Braak III-IV stages, no NFTs were detected in the visual cortex of either demented or resilient brains (Fig. 2c).

Total number of astrocytes, microglial cells and oligodendrocytes in the cortical and subcortical ROIs did not differ between control, resilient, and demented brains at Braak III-IV stages.

**Fig. 3:**
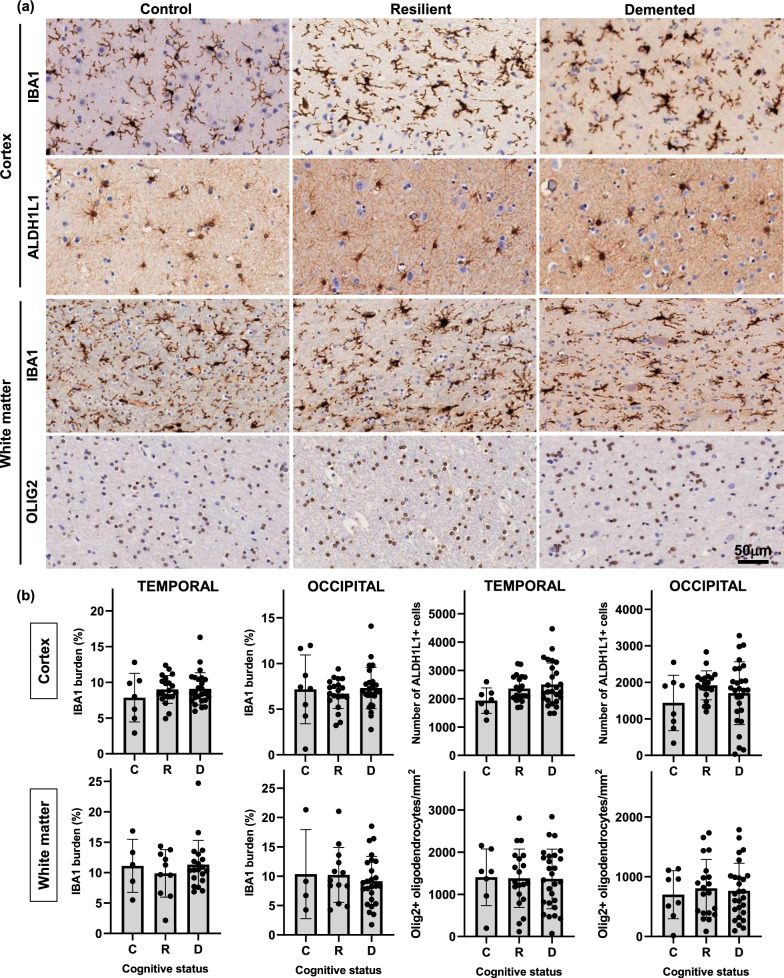
Quantifications for total IBA1 burden (percentage of cortex or white matter covered by IBA1 + microglia), total number of astrocytes (ALDH1L1), and total number of oligodendrocytes (Olig2) in temporal and visual cortex (microglia and astrocytes) and white matter (microglia and oligodendrocytes) (**b**) did not show statistically significant differences between demented, resilient and control brains. Representative photomicrographs of IBA1 + microglia, ALDH1L1 + astrocytes and OLIG2 + oligodendrocytes on sections from control, resilient and demented cases containing temporal pole are displayed on the top (**a**). *C* Control (Braak 0-II); *R* Resilient (Braak III/IV); *D* Demented (Braak III/IV). Scale bar 50 μm

Our results showed that total number and density of astrocytes (ALDH1L1), microglial cells (IBA1) and oligodendrocytes (Olig2), as reported by their respective constitutive markers in cortical and subcortical ROIs did not significantly differ between controls, resilient and demented groups (Fig. 3). These data are in agreement with previous reports [36] and suggest that phenotypic changes of existing resting glia rather than proliferation underlie glial responses in AD.

Phenotypic changes in glial cells are an early phenomenon that precede overt tau tangle pathology in demented individuals at Braak III-IV stages.

**Fig. 4:**
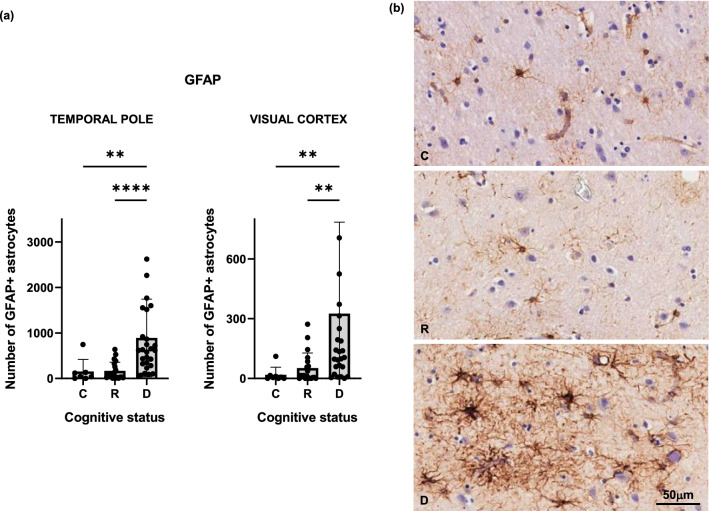
Number of reactive astrocytes as labelled by GFAP antibody were significantly higher in the temporal pole and visual cortex of demented but not resilient when compared to control brains (**a**). Representative photomicrographs of GFAP + astrocytes on sections from control, resilient and demented cases containing temporal pole are displayed on the right (**b**). *C* Control (Braak 0-II); *R* Resilient (Braak III/IV); *D* Demented (Braak III/IV); **p < 0.01; ****p < 0.0001. Scale bar 50 μm

Although total cell numbers remained stable, there was a phenotypic change in astrocytes in the temporal pole in the setting of dementia with a significantly higher number of GFAP + activated astrocytes compared to both controls and resilient brains (Fig. 4). Interestingly, a significantly higher number of GFAP + astrocytes was also observed in the visual cortex of demented compared to resilient brains despite absence of NFTs in this brain region and equivalent Aβ plaque burdens in both groups (Fig. 2).

**Fig. 5:**
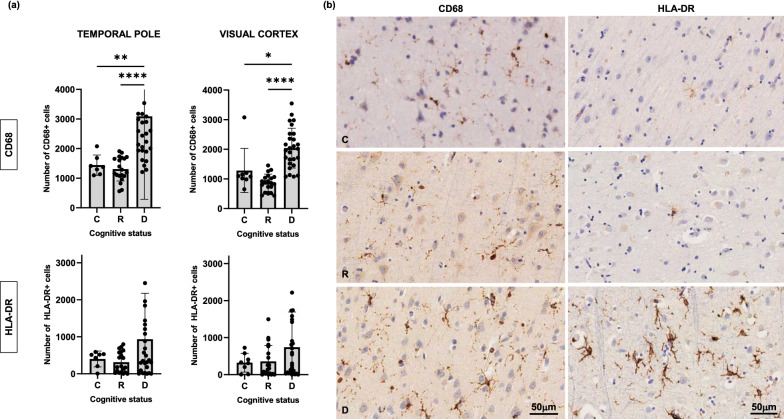
Number of CD68 + microglia was significantly higher in the temporal pole and visual cortex of demented but not in resilient when compared to control brains (**a**). A similar trend that did not reach statistical significance was observed in the number of HLA-DR + microglia (**a**). Representative photomicrographs of CD68 + and HLD-DR + microglia on sections from control, resilient and demented cases containing temporal pole are displayed on the right (**b**). *C* Control (Braak 0-II); *R* Resilient (Braak III/IV); *D* Demented (Braak III/IV); *p < 0.05; **p < 0.01; ****p < 0.0001. Scale bars 50 μm

**Fig. 6:**
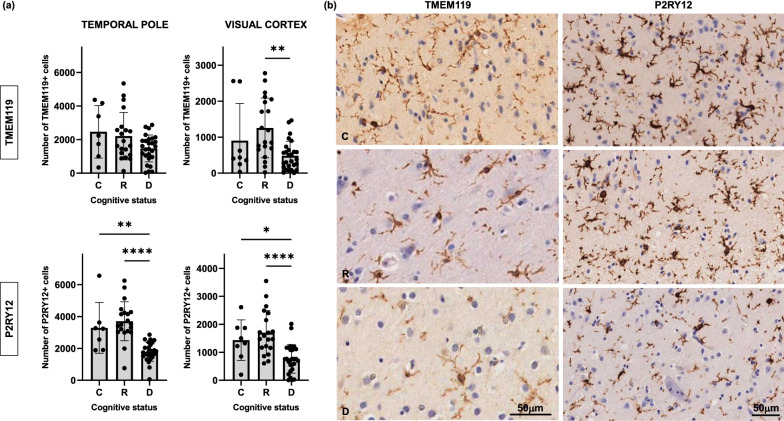
Homeostatic microglia stained with TMEM119 and P2RY12 antibodies in temporal pole and visual cortex was significantly decreased in the temporal pole and visual cortex of demented but not in resilient when compared to control brains (**a**). Representative photomicrographs of TMEM119 + and P2RY12 + microglia on sections from control, resilient and demented cases containing visual cortex are displayed on the right (**b**). *C* Control (Braak 0-II); *R* Resilient (Braak III/IV); *D* Demented (Braak III/IV); *p < 0.05; **p < 0.01; ****p < 0.0001. Scale bars 50 μm

A significantly higher number of pro-inflammatory microglial cells, as reported by CD68 antibody (Fig. 5), and a decreased number of homeostatic microglia, as reported by TMEM119 and P2RY12 immunolabeling (Fig. 6), were also found in the temporal pole of demented while no differences were found in resilient brains when compared to controls. Interestingly, similar changes in microglial phenotypes were found in the visual cortex of demented but not resilient individuals (Figs. 5 and 6) further suggesting that changes in glial phenotypes in demented individuals at Braak III-IV stages not only increase in parallel to tangle formation in regions already displaying tau aggregates (temporal pole) but also occur in advance of overt tau deposition in brain regions along the Braak tau pathway that are not yet impacted by tau pathology (visual cortex). Of note, no significant correlations were found between PMIs and pro-inflammatory (CD68 *p* = 0.40 and *p* = 0.51 in temporal pole and visual cortex respectively; HLA-DR *p* = 0.46 and *p* = 0.60 in temporal pole and visual cortex, respectively) or homeostatic microglial markers (TMEM119 *p* = 0.55 and *p* = 0.47 in temporal pole and visual cortex, respectively; P2RY12 *p* = 0.46 and *p* = 0.69 in temporal pole and visual cortex, respectively).

Whether these early glial phenotypic changes observed in visual cortex of demented individuals may set a path that facilitates subsequent pathogenic tau conversion/deposition and neuronal damage or represent a response to the presence of soluble pathogenic tau aggregates in Braak V-VI regions prior to overt tau tangle pathology remains uncertain [21]. It is possible that the absence of such aberrant glial cell responses in resilient brains may be capable of maintaining a homeostatic regulation of neuroinflammation and contribute to preserve neuronal cell integrity and thus brain function in these individuals.

Early markers of cellular DNA damage precede overt tau tangle pathology and correlate with phenotypic changes in glia of demented individuals at Braak III-IV stages.

**Fig. 7:**
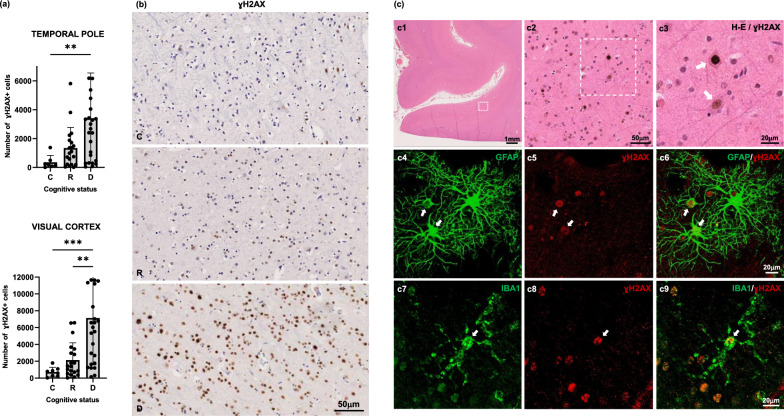
Number of γH2AX + cells was significantly increased in temporal pole and visual cortex of demented but not in resilient when compared to control brains (**a**). Representative photomicrographs of γH2AX + cells on sections from control, resilient and demented cases containing visual cortex (**b**). Representative immunofluorescence images of colocalization of γH2AX + cells (brown) with neurons (red) (7c 1–3, showing a progressively zoomed-in brain region), GFAP + astrocytes (green) (**c** 4–6, showing GFAP alone, 7c4, γH2AX alone, 7c5, both GFAP-γH2AX, 7c6), and IBA1 + microglia (green) (7c 7–9, showing IBA1 alone, 7c7, γH2AX alone, 7c8, both IBA1-γH2AX, 7c9). *C* Control (Braak 0-II); *R* Resilient (Braak III/IV); *D* Demented (Braak III/IV); **p < 0.01; ***p < 0.001. Scale bars 1 mm, 50 μm, and 20 μm. White arrows indicate colocalization between γH2AX+ cells and neurons, astrocytes, and microglia, respectively

Gamma H2AX (ɣH2AX) is a well-established biomarker of early cellular damage. Previous studies have shown that DNA damage resulting in double stranded DNA breakages initiates the phosphorylation of histone variant H2A at the Serine 139 site to generate ɣH2AX [81]. We quantified the number of ɣH2AX positive cells in the cortical ROIs of control, resilient and demented brains and found an increase in the number of ɣH2AX positive cells in the demented brains in both, temporal pole and visual cortex, but not in resilient brains (Fig. 7a, b). Importantly, a significant correlation between ɣH2AX and markers of cortical pro-inflammatory glia (GFAP, CD68, HLA-DR) were observed (Spearman R and p values for correlation analyses between cortical GFAP, CD68, HLA-DR and cortical ɣH2AX in temporal/visual regions: GFAP: R = 0.57/0.45 and *p* = 0.0006/ < 0.0001; CD68: R = 0.44/0.46 and *p* = 0.0009/0.0005; HLA-DR: R = 0.32/0.07 and p = 0.02/0.6, respectively).

Interestingly, the differences in the number of ɣH2AX+cells between demented and resilient were more pronounced in visual cortex than in temporal pole, demonstrating that cellular damage precedes overt NFT deposition, and pointing to the readily observed aberrant glial responses in the same brain regions as a potential contributor to early cellular vulnerability and brain dysfunction in demented individuals (Fig. 7a). Importantly, no significant correlation was found between number of ɣH2AX + cells and number of NFTs (*p* = 0.43 in temporal pole) or PMIs (*p* = 0.26 and *p* = 0.09 in temporal pole and visual cortex respectively). Of note, ɣH2AX immunolabelling was observed in neurons as well as in glial cells in demented brains (Fig. 7c) suggesting that both, neuronal and glial damage are part of the brain injury responses that ultimately result in functional changes and impaired cognition, recognized as the dementing disorder in AD.

Glial changes in subcortical white matter precede overt tau tangle pathology and correlate with markers of cortical cellular damage in demented brains at Braak III-IV stages.

The changes in glia within subcortical WM led us to examine whether changes in the subcortical glial phenotype could also be present at early tau pathological stages and predict cellular vulnerability and cognitive outcomes. We hypothesized that dysfunctional glial responses in juxtacortical WM tracts occurred prior to overt tau tangle deposition in the corresponding cortical regions and that they correlated with markers of early cortical cell damage in these brains.

**Fig. 8:**
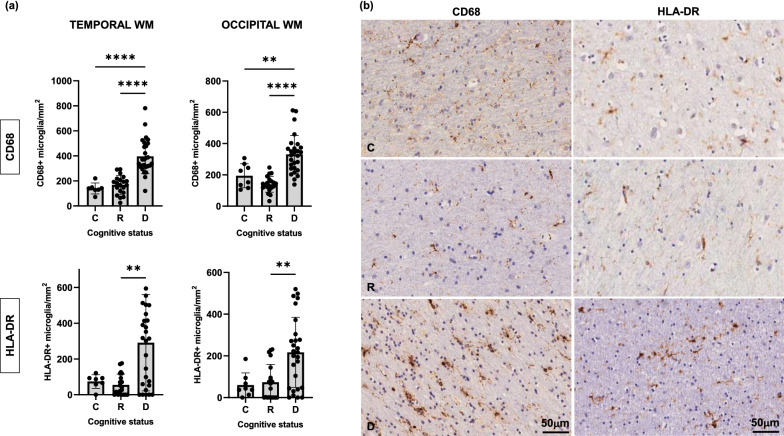
Density of reactive microglia (CD68+ , HLA-DR +) in temporal and occipital white matter (WM) was significantly increased in demented but not in resilient when compared to control brains (**a**). Representative photomicrographs of CD68+ and HLA-DR+ microglia on sections from control, resilient and demented cases containing temporal WM are displayed on the right (**b**). *C* Control (Braak 0-II); *R* Resilient (Braak III/IV); *D* Demented (Braak III/IV); **p < 0.01; ****p < 0.0001. Scale bar 50 μm

**Fig. 9:**
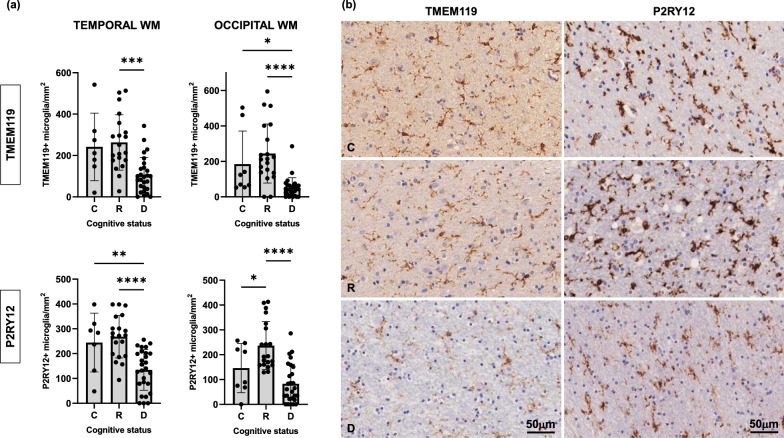
Density of homeostatic microglia (TMEM119 + , P2RY12 +) in temporal and occipital white matter (WM) was significantly decreased in demented but not in resilient when compared to control brains (**a**). Representative photomicrographs of TMEM119 + and P2RY12 + microglia on sections from control, resilient and demented cases containing temporal WM are displayed on the right (**b**). *C* Control (Braak 0-II); *R* Resilient (Braak III/IV); *D* Demented (Braak III/IV); *p < 0.05; **p < 0.01; ***p < 0.001; ****p < 0.0001. Scale bar 50 μm

In our analyses of juxtacortical WM we found a significant increase in activated pro-inflammatory microglia (CD68, HLA-DR) (Fig. 8) accompanied by a loss of homeostatic microglia (TMEM119, P2RY12) in WM of demented compared to resilient and control brains (Fig. 9). These WM changes were not only present in brain regions where cortical NFTs were already present (temporal pole), but also in areas without detectable tau burden (occipital cortex) suggesting that dysfunctional glial profiles in WM are present early in the disease course and precede overt tau deposition in the overlying brain cortices. In agreement with the results above, we also found that total number of oligodendrocytes (Olig2) and total microglial (IBA1) cell numbers and burden in WM remained unchanged across groups regardless of presence and/or absence of tau deposition in the immediately adjacent cortex (Fig. 3), further reinforcing the idea of qualitative rather than quantitative glial changes in demented AD brains.

Because the clinical symptoms characterizing the dementing disorder of AD are predominantly cortical rather than subcortical in nature, we evaluated whether the subcortical glial changes observed were associated with cortical cellular vulnerability (ɣH2AX). Interestingly, we found a significant inverse association between number of TMEM119 + microglial cells in the WM and number of ɣH2AX + cells in immediately adjacent temporal and visual cortical regions (Spearman R and p values for correlation analyses between WM TMEM119 and cortical ɣH2AX in temporal/visual regions: R =  − 0.36/ − 0.29 and *p* = 0.007/0.03 respectively), which did not reach significance when correlating TMEM119 and ɣH2AX positive cells in the cortex. This suggests that loss of homeostatic microglia in the WM is an early brain change that occurs in advance of classic tau tangle pathology and may potentially contribute to cortical cell damage and loss of brain function in demented brains. It is also possible that the higher burden of overall cerebrovascular risk observed in the demented individuals might be contributing to early glial derangements in the WM of these individuals even in the absence of significant differences in vascular insults across groups. Of note, identical changes in cortical and subcortical glial phenotypes and their correlation with markers of cortical cell damage were also present when analyses were limited to the subset of individuals with PART (not shown).

## Discussion

In this study we have found that early changes in the phenotype of glial cells in the cortex and white matter (WM) – involving both increased pro-inflammatory and decreased homeostatic responses – occur in advance of overt NFT formation along the pathologically defined tau pathway, strongly correlate with markers of cortical cell damage, and more accurately predict than tangles themselves the cognitive status of individuals who are at tau Braak III-IV stages at autopsy. These results favor a model where early glial dysfunction in cortical and subcortical regions may be a key element of the tissue injury response that determines the cognitive fate of individuals who harbor intermediate stages of classic AD tau tangle pathology in their brains. Our data also suggest that the absence of such aberrant glial cell responses in resilient brains may reflect maintenance of homeostatic regulation of neuroinflammation and contribute to the preservation of cell integrity as a mechanism for resilience. If true, this would highlight possible new targets for intervention in the face of plaques and tangles.

Current schemes for assessing disease burden in AD examine the distribution, abundance and characteristics of plaques and tangles, yielding an estimate of the likelihood of cognitive impairment. While this is highly predictive for most individuals, in some instances there is a mismatch between lesions and symptoms. Some individuals harbor a substantial burden of these lesions at autopsy which would be expected to have had deleterious consequences on their brain function, but remain at their cognitive baseline. This phenomenon of absence of overt cognitive deterioration in the face of neuropathologic lesions expected to have had substantial clinical consequences is the process we and others have termed ‘resilience’ to AD pathology (and we refer to the tissue from such individuals as ‘resilient brains’). While resilient brains with a high burden of AD pathology meeting criteria for high probability of AD at autopsy (moderate-frequent neuritic plaques and tangle Braak stage V-VI) according to NIA-AA guidelines [39] represent only a small subset of outliers [18, 68], the percentage of cognitively unimpaired individuals meeting an intermediate probability of AD at autopsy (moderate-frequent neuritic plaques and tangle Braak III-IV stage) is substantially higher at around 40% [63, 64]. This evident disconnect between dementia status and classic AD neuropathologic changes (e.g. plaques and tangles) [38] sets the stage for investigating additional brain changes that could be meaningful to more accurately predict at the individual level the presence or absence of dementia in the setting of amyloid and tau lesions.

Trying to understand the relationship between plaques, tangles and cognition can be confounded by a wide range of variables, including individual differences in the regional burden of lesions [38], genetic risk and protective factors [61], presence of distinct tau strains with different propensity to induce tau aggregation [22], as well as life style factors including educational attainment [8] and cognitive activity across the life span [99], that likely contribute to interindividual variation. Moreover, there is the complicating impact of other concomitant neurodegenerative processes (including those marked by inclusions containing alpha-synuclein and TAR DNA-binding protein 43 (TDP-43)) and vascular brain lesions (including small vessel disease such as arteriolar sclerosis and cerebral amyloid angiopathy (CAA), as well as stroke) whose prevalence also increases with age [6, 25, 46]. Clinical-pathological correlation analyses demonstrate that the burden of these lesions lower the threshold for the clinical diagnosis of dementia in the face of low levels of classic AD neuropathological changes [45, 74], yet a recent study showed that over one third of cases with clinically manifest AD cannot be attributable to all these age-related neuropathologic indices (AD pathology and comorbidities) combined [12]. We and others have previously demonstrated that even after excluding individuals with significant concomitant neurodegenerative and vascular lesions and carefully matching burden and regional distribution of plaques and tangles, there is a small subset of individuals who appear capable of tolerating the insult of a full burden of plaques and tangles (moderate-frequent neuritic plaques and tangle Braak V-VI stage) in their brain without developing dementia [18, 68, 73, 77]. Intriguingly, the study of these unique brains revealed that distinct glial cell responses and brain cytokine expression profiles may serve to more accurately predict future brain cell damage and cognitive fate than plaques and tangles themselves [5, 56, 68], pointing to neuroinflammation as a potentially significant contributor to cell damage and loss of brain function in AD rather than an innocent bystander response to neuronal injury.

In keeping with the above literature, the present study primarily focused on detailed assessments of glial phenotypes of brains from individuals who harbor intermediate stages of NFT pathology at autopsy (Braak stage III-IV) but exhibited opposite antemortem clinical outcomes (demented vs. cognitive normal `resilient’). It is well known that tangle pathology appears in human AD brains in a hierarchically spatial consistent manner, starting in the transenthorinal/entorhinal cortex (Braak stage I-II), and ultimately culminating in the primary visual cortex (Braak stage VI), resulting in overt tau pathology and cell damage along the same pathway. Thus, intermediate Braak stages (Braak stages III-IV) provide a unique window of opportunity to investigate other brain changes occurring in later regions in the Braak tau pathway (visual cortex) that precede tau tangle accumulation and may ultimately drive, rather than tangles themselves, neurodegeneration and change from baseline function.

Importantly, brains with comorbidities like TDP43 and alpha-synuclein were excluded from the present study to avoid additional potential confounders contributing to the heterogeneity in the clinical expression of tangle Braak III-IV stages. We confirmed that the regional burdens of both plaques and tangles in the ROIs studied (temporal pole, Brodmann’s area 38, and visual cortex, Brodmann’s area 17/18) were well matched between demented and resilient brains thus ruling out the most parsimonious explanation that a higher regional load of lesions in the demented could explain the divergent clinical expression of the two groups. Even though multiple studies, including our own, have shown that the number of NFTs tends to correlate much better than Aβ plaques with the severity and duration of clinical symptoms and the amount of neuronal loss [3, 28], some neuronal populations are vulnerable to cell loss with limited tangle formation [11]. Even in areas that develop abundant tangles, the vast majority of neuronal loss in patients with clinically manifest AD cannot be explained by the number of tangles present at autopsy [28, 29, 92]. Moreover, in mice overexpressing mutant tau to mimic AD neuronal loss, tangles on their own are not sufficient to permanently disrupt cognitive function and, similarly to human AD, the extent of neuronal loss in their brains exceeds by far tangle numbers [76]. Increasing evidence from mouse models and human brains supports a role of soluble pathological tau assemblies leading to synapse and neuronal damage [50]. We and others have previously observed a robust aberrant accumulation of soluble species of hyperphosphorylated tau within the synaptic compartment in clinically manifest AD brains compared to very small quantities or absence of those in resilient brains with comparable tangle burdens [68, 84], suggesting that soluble pathological species of tau, rather than tau tangles, might be the critical toxic moiety leading to neurodegeneration and clinical expression of AD. So even though amyloid and tau are likely instigators, other downstream mechanisms must be the real culprits driving brain cell damage and dementia in individuals who harbor these proteins in their brains.

Previous studies have convincingly shown that activation of astrocytes and microglial cells are present early and linearly increase around plaques and tangles with disease progression in clinically manifest AD brains [80]. Additionally, recent studies have also identified a loss of homeostatic glial gene expression that can exert detrimental effects in early steps of AD pathogenesis [62, 85]. In the present study, we used a panel of well characterized antibodies against constitutive as well as pro-inflammatory and homeostatic glial markers. GFAP is the main intermediate filament protein found in astrocytes and gets upregulated upon abnormal astrocytic activation. In AD, it has been found to increase from mild cognitive impairment (MCI) stages and to be particularly expressed in the vicinity of Aβ deposits [15]. In addition, recent studies have found higher GFAP levels in cerebrospinal fluid and plasma of clinically symptomatic AD patients, and an association between plasma GFAP and a higher burden of WM lesions and degree of cognitive impairment [66]. Activated proinflammatory microglia is characterized by increases in CD68, a lysosomal associated membrane protein (LAMP) that gets upregulated upon increased phagocytic activity, and HLA-DR (human leukocyte antigen class II molecule), a membrane bound glycoprotein used by antigen-presenting cells to expose fragments of phagocytosed pathogens and trigger additional inflammatory responses [95]. Studies of postmortem human brains have shown that both, CD68 and HLA-DR, robustly increase in microglia of demented AD brains across brain regions [36], and that these effects go beyond the pro-inflammatory state observed in normal ageing [43]. Although much of microglial characterization in AD has focused on the overexpression of pro-inflammatory genes, recent studies have also identified that loss of homeostatic microglial genes can exert detrimental effects in early steps of AD pathogenesis [62, 85]. Loss of the homeostatic phenotype of microglia is in fact a hallmark of proinflammatory states in general and a key element across various neurological conditions [47, 100]. Interestingly, a recent study found a significant association between the amount of neuronal loss and the degree of reduced homeostatic microglial gene expression profiles in AD mouse models and in RNAseq analyses of the precuneus in human brains [85]. To date, the most specific microglial homeostatic markers are TMEM119 and P2RY12 [43]. While the function of TMEM119, a transmembrane protein that distinguishes resident microglia from blood-derived macrophages, remains largely unknown, P2RY12 is involved in sensing external injury signals through its purinoreceptor and in chemotaxis upon stimulation through external injury signals. We found a distinct change in the phenotype of glial cells in demented brains at Braak III-IV stages with significantly increased pro-inflammatory astrocyte and microglial markers (GFAP, CD68), and decreased homeostatic microglial markers (TMEM119, P2RY12) that distinguished them from age-matched resilient and control brains. Importantly, these glial phenotypic changes in demented brains were present not only in brain areas that already contained NFTs at Braak III-IV stage (temporal pole), but also in areas with no overt tangle formation by immunohistochemistry (visual cortex). Identical glial phenotypic changes were found in demented in the adjacent subcortical WM in the same brain regions but not in resilient or control brains. Moreover, both grey and white matter glial cell abnormalities in demented significantly correlated with an increased expression of ɣH2AX, a marker of early cellular damage, both in neurons and glia. These novel insights favor a model where glial responses, and in particular microglia—the immunocompetent cells of the central nervous system—and immune-related mechanisms may be real culprits of cell injury and impaired brain function in the setting of plaques and tangles. Of note, after matching the three groups for cerebrovascular pathology, demented brains still showed an overall higher composite cerebrovascular risk score when compared to resilient and control brains. All these observations are in agreement with previous findings from radiological and genetic studies that point to the contribution of elevated vascular risk biomarkers and genetic variants that promote inflammation to age-related cellular/vascular changes in the cerebral WM (considered to be the substrate of WM hyperintensities) and increased risk and faster progression of cognitive impairment in the face of classic AD pathology (e.g. plaques and tangles) [67, 69, 71].

This study is not without caveats. First, while observations derived from autopsy studies like the present one may allow to infer information about the hierarchical order of pathological brain changes, their temporo-spatial relationships, and the resulting impact on antemortem brain function, the cross-sectional nature of such studies precludes drawing conclusions on specific underlying molecular mechanisms involved. Secondly, we cannot rule out the possibility that distinct tau strains may exist in demented and resilient brains with different biological properties including their propensity to form toxic soluble tau species (tau seeding activity) prior to the development of overt tau pathology, or the intensity of phosphorylation of different tau phosphor-epitopes that may result in their differential glial cell responses and clinical disease expression. Alternatively, the possibility also exists that the aberrant early changes in the glial phenotype in the grey and white matter of demented brains that precede tangle formation may be the real culprits of neuronal and glial damage and impaired brain function, and that preservation of physiological phenotypes of astrocytes and microglial cells would be needed for a human brain to cope with the challenges of Aβ and tau aggregation and deposition and thus preserve its normal function. Future studies will be conducted to address these important questions.

In sum, the present study demonstrates that loss of homeostatic phenotypes of microglia and rise in pro-inflammatory astrocytes and microglial cells in the cortex and WM precede NFT formation along the well-defined tau pathological pathways in demented individuals who are at intermediate stages of classic tangle burden (Braak stages III-IV). These changes in glial phenotypes closely correlate with early markers of DNA damage in neurons and glia and accurately distinguish demented individuals from those exhibiting preserved cognition at a similar tau Braak stage at autopsy. These novel findings have potential important implications to guide the development of new biomarkers and also to develop novel therapies that can prevent cognitive impairment in the presence of classic AD lesion burden (e.g. plaques and tangles). The development of surrogate in vivo markers capable of detecting early changes in the glial phenotypes and signs of early neuronal and glial vulnerability in advance of the predicted spreading of tau aggregation between anatomically connected brain regions has the potential to more precisely identify who will develop clinical symptoms of dementia among asymptomatic amyloid and tau positive elderly at intermediate stages of pathology and over what time frame, providing valuable guidance on the need and optimal timing for personalized preventive intervention.

## Data Availability

Original slides and diagnostic material are retained. There are no novel reagents or materials for others to request.
